# Evolving Proteomic Biomarkers in Children with Congenital Heart Defects

**DOI:** 10.3390/jcdd13080360

**Published:** 2026-08-01

**Authors:** Martin Schweiger, Clemens Haselmann, Robert Cesnjevar

**Affiliations:** 1 Division of Cardiac and Vascular Surgery, University Children’s Hospital Zurich, 8008 Zurich, Switzerland; 2 Cardiac Surgery, Heart Center, King Faisal Specialist Hospital & Research Center, Riyadh 13338, Saudi Arabia; 3 Children’s Research Centre, University of Zurich, 8008 Zurich, Switzerland; 4 Department of Cardiac Surgery, University Hospital Erlangen, Friedrich Alexander University Erlangen-Nürnberg, 91054 Erlangen, Germany

**Keywords:** congenital heart disease, cardiac biomarkers, BNP/NT-proBNP, Galectin-3, sST2, P3NP, Growth differentiation factor-15, copeptin

## Abstract

Conventional biomarkers provide useful information but often lack specificity due to age-related physiological variations or in the context of congenital heart disease (CHD). Proteomics uses new technologies to analyze hundreds to thousands of proteins simultaneously (multi-biomarker panels) and, combined with traditional ones (i.e., NT-BNP), it improves risk prediction and helps clinical decision making. In adults, there are clear efforts to improve risk prediction accuracy and personalized HF management strategies; meanwhile, more evidence must be established in the pediatric CHD population. This non-systematic review aims to give an overview of the limitations of conventional biomarkers and recently published literature on promising evolving biomarkers of the proteomic family that are applicable to the CHD population. Due to the highly complex physiology and interpretation of the anatomical variation of the biomarkers, it seems to be necessary for single markers to be tested in the pediatric population with various CHD anatomies and physiologies. Disease-specific validation is required, rather than assuming they behave the same way as they do in structurally normal hearts and adults.

## 1. Introduction

Advances in the surgical and medical field have improved treatment options and outcomes for children born with congenital heart disease (CHD). This has led to a growing population of patients living with CHD. These patients remain at risk for heart failure (HF), arrhythmia and/or pulmonary hypertension (pHT).

Proteomic biomarkers are measurable proteins or protein patterns in the blood of the patient. These proteins are the molecules most directly responsible for cell damage. In HF, myocardial tissue releases proteins related to fibrosis, myocardial stretch, cell injury, oxidative stress or neurohormonal activation. Several proteins act as biomarkers, as their levels change in relation to the amount of tissue damage. The best known and most clinically important proteomic biomarkers in cardiac diseases are natriuretic peptides, including B-type natriuretic peptide (BNP) and N-Terminal pro-B-type natriuretic peptide (NT-proBNP), as well as cardiac troponins such as high-sensitive Troponin I and T.

These conventional biomarkers are well studied, well established over decades, and represent the gold standard. They provide useful information in terms of helping to identify high-risk patients and perform risk stratification, and they may guide further treatment options. They have also been proven to play a crucial role in the non-invasive assessment of cardiac function and in the management of acute and chronic HF in multiple international guidelines [1,2,3].

While these conventional biomarkers provide useful information, they often lack specificity due to age-related physiological variations or in the context of CHD. In recent years, novel proteomic biomarkers have evolved and been tested and established in adults. There is increasing interest in the testing and evaluation of these biomarkers in pediatrics and the fast-growing population of adults suffering from CHD.

This non-systematic review aims to give an overview of the limitations of conventional biomarkers and recently published literature on promising evolving biomarkers of the proteomic family that are applicable to the CHD population.

A comprehensive literature search was conducted in MEDLINE via PubMed, including studies published from 1 January 2015 to June 2026. The search strategy was a free-text keyword search including search terms: congenital heart disease, proteomic biomarker, pediatric populations, heart failure, cardiac surgery, adult congenital heart disease, and biomarkers known to the reviewers in the adult field. Reference lists of included studies and relevant reviews were manually screened to identify additional eligible studies. Two researchers reviewed studies and selected publications according to relevance to CHD population. The exclusion criteria were as follows: if there were non-CHD diseased patients mentioned, or the full article was not in English.

## 2. Challenges of Biomarkers in CHD

Within the CHD population, there are several barriers to testing and interpreting cardiac biomarkers. It is not a homogenous group, including newborns, infants and babies, but also grown-up teenagers and adults. Normal reference ranges differ significantly across developmental stages. Besides this age-related challenge, the other major challenge is the heterogeneity of CHD itself. Patients have vastly different anatomical abnormalities and physiological adaptations, i.e., cyanotic CHDs, patients with shunt lesions, or the unique group of single ventricle patients. This makes it difficult to transfer thresholds from the non-CHD adult population to patients diagnosed with and treated for CHD [4]. For example, Fontan patients have a non-physiologic circulation with chronically elevated venous pressures and liver congestion, as well as different renal perfusion patterns. This affects biomarker production, metabolism and clearance. Additionally, surgical interventions and long-term adaptations (such as ventricular remodeling) can alter biomarker expression independently of current clinical status.

For all these reasons, it is understandable that most of the published studies on cardiac biomarkers exclude patients below 18 years of age, as well as patients diagnosed with CHD. Researchers want a “clean” population first to determine whether a new biomarker truly reflects the disease being studied. Including CHD patients will increase variability and therefore reduce statistical power. CHD patients have persistent myocardial stretch, lack ‘normal’ saturations, and cope with hypoxemia; additionally, biomarker levels, i.e., troponin and BNP/NT-proBNP, may already be elevated at baseline, making it difficult to determine whether changes are due to the condition under study or the CHD itself.

## 3. Conventional Biomarkers and Their Limitations

The precursor of BNP that was first isolated in porcine brain cells [5,6], known as pre-pro-BNP, is synthesized by myocardial cells caused when they are stretched. It is subsequently cleaved by two enzymes, leading to an active form and an inactive peptide. The inactive one is known as NT-proBNP. This marker has significantly improved the assessment and management of HF patients [7] and has become one of the best established and globally widely used biomarkers.

Evidence for its use in children is not as high as in adults. First, the natural course of NT-proBNP levels shows that their levels are high straight after birth and then decline over the following weeks to months [8,9]. Adult cutoffs might therefore be unreliable, especially in neonates, infants and young children. While the data show diagnostic utility in children [10], no clear, fully standardized range has been established for them [9,10]. There are age-specific references published [11,12], but it must be noted that most of the studies were condition-specific and conducted in pediatric HF patients [13]. Other known limitations of BNP/NT-BNP are elevated levels when there is reduced renal clearance [14]. Levels might be high in infections (sepsis, systemic inflammation, pneumonia) but also when there is pHT. Left to-right shunts in CHD patients may lead to pHT. Wang et al. studied CHD patients with a left to right shunt and found elevated levels of NT-proBNP [13].

On the other hand, there is good evidence that NT-proBNP and BNP have their uses in CHD patients, especially adult CHD patients. Data indicate that NT-proBNP can function as an independent predictor for HF in children with CHD [15], especially when combined with diagnostic criteria such as the modified Ross criteria [10]. A recently published study in pediatric cardiac transplantation reported that NT-proBNP values are likely a surrogate for HF [16]. Likewise, NT-proBNP is the most studied blood test in Fontan patients [17]. Li et al. found that NT-proBNP is a very useful marker for mid- and long-term monitoring after Fontan surgery, and the levels correlate with heart function and clinical outcomes [18]. There also seems to be similarities between adult post-Fontan patients and adults who have acquired HF with preserved (HFpEF) ejection fraction [19]. It was reported that NT-proBNP and BNP are strong independent predictors of clinical events in patients with HFpEF [20]. For the group of single ventricles, Palm et al. showed that age-adjusted NT-proBNP levels were significantly higher in patients with a systemic right ventricle compared to a systemic left ventricle [21].

## 4. Emerging Novel Biomarkers for the CHD Population

Out of the proteomic family, a few emerging markers (Galectin-3 (Gal-3), soluble ST2 (sST2), Procollagen Type III N-Terminal Propeptide (P3NP), Growth differentiation factor-15 (GDF-15) and Copeptin) are covered in CHD patients published studies and should be discussed in more detail.

## 5. Galectin-3 (GAL-3)

Galectin-3 (Gal-3) belongs to the galectin family of lectins and, unlike natriuretic peptides, which primarily reflect myocardial stretch, Gal-3 reflects fibrosis and chronic remodeling. This β-galactoside-binding lectin is produced by activated macrophages and fibroblasts. Gal-3 also plays a role in cellular adhesion, apoptosis and immune activation [22]. In a recent study in which healthy male adults performed high-intensity interval training, Gal-3 was associated with acute exercise-induced endothelial activation [23]. Other studies showed that Gal-3 can be used as a marker for HF severity [24] and mortality risk [25,26]. In adult Fontan patients, elevated plasma Gal-3 levels (median 11.85 ng/mL) were observed, and these patients had an increased risk of nonelective cardiovascular hospitalization or death [27]. The FDA approved Gal-3 for risk stratification in HF patients.

Most data pertaining to GAL-3 have been reported in children with HF symptoms rather than specific CHD diseases. There are some data suggesting that Gal-3 may contribute to early diagnosis, prognosis, and therapeutic monitoring in children suffering from CHD [28,29]. Elevated levels have been observed in pediatric patients with HF and may correlate with disease severity [28,29,30]. A prospective study by Saleh et al. evaluated Gal-3 in children with CHD-associated HF and showed significantly elevated serum Gal-3 concentrations in symptomatic patients compared with asymptomatic CHD patients and healthy controls. Gal-3 levels correlated positively with Ross heart failure classification and echocardiographic indices of ventricular dysfunction [30]. Hoshino et al. reported elevated Gal-3 levels during active Kawasaki disease, particularly in patients with coronary artery involvement and a low ejection fraction [31].

Because Gal-3 is secreted by activated macrophages and immune cells, it has also emerged as a marker in sepsis. This overlaps with inflammatory and renal diseases [32], which reduces specificity. Further age-related physiological variability complicates the interpretation of Gal-3. There are no standardized pediatric reference values so far.

## 6. Soluble ST2 (sST2)

ST2 (suppression of tumorigenicity 2) is a member of the interleukin-1 receptor family and exists in two main forms. One is bound to the membrane (transmembrane form) and is believed to be cardioprotective via an interaction with IL-33. The second form is the circulating soluble ST2, which acts as a decoy receptor, binding IL-33 and blocking its beneficial effects. Its concentrations are believed to reflect cardiovascular stress, and persisting elevated levels may indicate loss of cardioprotective signaling. A recent meta-analysis of 17 studies in adult chronic HF patients reported an increased all-cause mortality (HR 1.03), an increased hospitalization rate (HR 1.50) and higher likelihood of HF readmission (HR 1.46) with elevated sST2 levels in the blood [33]. Another prospective cohort study of adult patients with HFrEF revealed that Patients with sST2 > 35 ng/mL had a significantly higher one-year cardiovascular mortality and rehospitalization rate. The authors concluded that a lack of reduction in serial sST2 levels predicted poor composite outcomes [34].

Emdin et al. reported that sST2 is an independent predictive value for all-cause and cardiovascular mortality, as well as for hospitalization in chronic HF [35]. The authors suggested that it should be part of a multimarker panel, together with NT-proBNP and hs-TnT. In the 2013 American Heart Association/American College of Cardiology HF guidelines, sST2 is mentioned as a prognostic biomarker for risk stratification but not as a primary diagnostic marker [36]. In the revised 2017 ACC/AHA/HFSA, “Focused Update Guidelines for the Management of Heart Failure”, ST2 was given a class IIa recommendation for the optimal risk assessment in patients with HF [37].

There are some data related to adults suffering from CHD that indicate sST2 might have additional prognostic value. The study group of Laqqan investigated the biomarker in 169 consecutive adult patients with complex CHD. They concluded that it may have additive value for natriuretic peptides for the prediction of all-cause mortality [38]. Additionally, in adults suffering from pHT, it proved to be a strong prognostic marker [39,40]. For the (adult) Fontan population, some data exist (see below).

Unlike natriuretic peptides, sST2 is believed to be less strongly influenced by body mass index, renal function and age. This might make it a valuable tool in pediatric CHD patients. In children, a study evaluated baseline references for sST2 concentrations in healthy individuals. Serum sST2 concentrations seemed to be stable across age groups aged 2–17 years and across sex. Reference intervals were set, ranging from 9 to 50 ng/mL. [41]. In pediatric patients with dilated cardiomyopathy, serum sST2 levels were associated with adverse outcomes and have prognostic value [42]; it could accurately discriminate between patients with preserved and patients with poor functional [43]. CHD-specific disease data on sST2 is rare. Sulu et al. found higher sST2 levels in children with valvular regurgitation and with growth retardation [39]. A few studies reported elevated plasma levels of sST2 in adult Fontan patients [44] also associated with severe adverse events or HF [38,45]. In contrast, the study group of Behnke et al. found that sST2 performed poorly in children with CHD and, when used in conjunction with NT-proBNP, it did not add significantly to its diagnostic accuracy [43]. One of the limitations seems to be specificity, as elevations may also occur in inflammatory or systemic diseases.

## 7. Growth Differentiation Factor-15 (GDF-15)

Growth Differentiation Factor-15 (GDF-15) is a stress-responsive cytokine belonging to the Transforming Growth Factor-beta (TGF-β) superfamily [46]. It is normally expressed at low levels but increases with ischemia, pressure overload or inflammation [47]. In recent years, published literature on adults has shown that GDF-15 levels are associated with HF, adverse cardiovascular events and adverse cardiac remodeling [48,49,50,51]. Already in 2007, Wollert et al. were able to show that GDF-15 is a marker of risk for death in patients with non-ST-elevation acute coronary syndrome [52]. In a large prospective study of post-acute myocardial infarction patients, elevated levels independently predicted mortality and HF, even after adjustment for traditional risk markers such as NT-proBNP [52]. In systematic reviews and metanalysis, increased concentrations of GDF-15 are associated with higher risks of hospitalization, rehospitalization, and cardiovascular mortality [53,54,55].

In operated ACHD patients, elevated GDF-15 levels were associated with impaired cardiac function and reduced exercise capacity, indicating its potential role in identifying patients at risk of developing HF [56]. In children suffering from CHD, Paneitz et al. showed that there are higher plasma GDF-15 concentrations (613–1628 pg/mL), particularly in those younger than two years and in defects associated with congestive HF. The study suggested that GDF-15 may reflect the metabolic and inflammatory burden associated with severe congenital cardiac disease [57]. In this study, the marker was also tested in different CHD-specific diseases including Tetralogy of Fallot, VSD, ASD, single ventricle and AVSD. The highest levels were observed in patients with AVSD (median 2244 pg/mL).

In a subset of children with dilated cardiomyopathy, GDF-15 was associated with poor left ventricular function and was thought to accurately discriminate between children with preserved or poor function [41].

Despite these promising findings, GDF-15 remains a non-specific biomarker because its levels may also rise in renal dysfunction, infection, malignancy, and systemic inflammatory states. Additionally, assay standardization and clinically validated cut-off values remain incomplete. Current evidence therefore supports GDF-15 primarily as a prognostic rather than diagnostic biomarker [58].

## 8. Copeptin

The arginine–vasopressin system plays a crucial role in the regulation of the individual endogenous stress response [59] and levels of arginine–vasopressin have been shown to be elevated in HF [60]. Copeptin is a fragment of the precursor peptide provasopressin, which is released in equimolar amounts with arginine–vasopressin. In contrast to arginine–vasopressin (half-life: 5 to 15 min), copeptin is stable in plasma and can be measured reliably [61]. Like MPO, because of this rapid release, copeptin levels rise very early when myocardial infarction occurs—often before cardiac troponins become detectable [62]. It has been studied as an adjunct biomarker for early rule-out of AMI [63]. When used with cardiac troponins, it improves the early diagnostic sensitivity [64,65]. Like other biomarkers copeptin reflects neurohormonal activation in HF and elevated levels are associated with higher hospitalization rates [64] and increased mortality [65]. Several studies suggest that copeptin provides prognostic information independent of BNP or NT-proBNP and may improve risk stratification when used in combination with natriuretic peptides [66]. Despite promising results, its drawbacks include low disease specificity, because it rises in many acute illnesses including sepsis and stroke, and variable cutoff values between published studies [67,68].

In the CHD landscape, elevated plasma copeptin levels have been reported in children with pHT [69]. It was found to be a good predictive marker for the severity of pHT and marks poor prognosis in these children [69]. Furthermore, copeptin levels were positively correlated with hypertensive children and obesity [70]. The marker was tested in children suffering with cardiomyopathy. In this population, it was associated with the severity of HF and adverse outcomes. Studies reported levels of 25 pg/mL or higher with worse outcomes [71,72].

## 9. Procollagen Type III N-Terminal Propeptide (P3NP)

Procollagen Type III N-Terminal Propeptide (P3NP) is a collagen-III-derived product that is released into the circulation. Deposition of PIIINP is a component of the myocardial extracellular matrix and an integral feature of cardiac remodeling [73,74]. The marker reflects ventricular dysfunction and adverse outcomes, particularly in patients with chronic pressure or volume overload. Studies evaluating PIIINP in pediatric CHD have demonstrated increased circulating levels in patients with both pressure- and volume-overload lesions, such as Tetralogy of Fallot and VSD/ASDs [75]. Sugimotot et al. conducted a trial including 172 patients with single-ventricle circulation and 149 controls; it elevated levels of P3NP in patient with a single ventricle [76]. The highest levels of P3NP the authors found was in patients with a BT shunt with levels of 0.604 U/mL. The PIIIP levels decreased with advancing surgical stages as ventricular volume load and cyanosis were alleviated [76].

In adult HF patients, P3NP was reported to be a marker for evaluating the risk of sudden cardiac death. A study of 84 pediatrics with CMP compared with healthy controls showed that there was a significantly higher plasma levels of total P3NP with a cut-off levels of 3 mg/L [77].

The limitations are that P3NP is not a sole myocardial fibrosis marker but more of a general fibrosis marker, as it was also associated with hepatic, pulmonary fibrosis, renal dysfunction, and with connective tissue disorders. The major limitation in children might be its strong dependence on age and physiological growth [78,79].

## 10. Discussion

Technological developments and advancements open up new possibilities for the biomarkers used in the detection and treatment of HF. Over the last decade, in addition to well-established cardiac biomarkers, new ones have evolved and have been increasingly used. The aim of this review was to give an overview of biomarkers of the proteomic family that are applicable to the CHD population and their mechanism of action. NT-proBNP and BNP still represent the gold standard and have been justified in adult CHD patients. This is best reflected by their appearance in the 2025 ACC/AHA/HRS/ISACHD/SCAI Guideline for the Management of Adults with CHD [80].

Proteomics uses new technologies to analyze hundreds to thousands of proteins simultaneously (multi-biomarker panels). Issues such as universal standardization across assays and the possibility of widely available tests in routine clinical settings must be investigated.

While the attempt of combining novel biomarkers with traditional ones is clear, cutoff values for specific CHD lesions or groups must be tested. When analyzing the results of this review, we were only able to find some tested reference intervals for some of the biomarkers (sST2, GDF-15). The validation of cutoff values for CHD-specific disease for most of these novel biomarkers is lacking. Only for copeptin was a cutoff value of 25 pg/mL in children suffering from dilated cardiomyopathy found.

In this review, we were able to show that most markers are tested for HF. Biomarker profiles for specific CHDs or groups (i.e., cyanotic vs. non-cyanotic, shunt lesions) are missing. Single emerging markers should be tested in the pediatric population with various CHD anatomies and physiologies. It requires disease-specific validation rather than assuming they behave in the same way as they do in structurally normal hearts. A key goal for translating novel biomarkers into routine practice seems to us to establish age- and lesion-specific reference ranges. Likewise, it is obvious that Fontan failure must be analyzed separately from biventricular failure in CHD patients. The limitation of reaching significant numbers for subgroup analysis must be overcome with multi-institutional trials.

Most available studies have measured biomarkers during early or late postoperative follow-up, sometimes perioperatively but so far not continuously during surgery. Interest is growing in biomarkers used continuously during surgery. The future direction of research will involve moving beyond simply detecting HF and measuring myocardial injury during congenital heart surgery. This could provide real-time or near-real-time information about myocardial function, tissue perfusion and inflammation. Novel biomarkers could add widely to existing biomarkers that are integrated into clinical practice for congenital heart surgery, e.g., serum lactate. It would open the door to improving the guiding of intraoperative management (i.e., perfusion strategy, medical treatment during surgery) and to identifying complications early. This could improve postoperative outcomes.

In the new area of integrating artificial Intelligence into clinical practice, combining biomarker profiles with echocardiography findings and cardiac MRI results in machine learning models have significant potential, especially in pediatric patients. It might not only give us a better understanding of the long-term prognosis of the children suffering from CHD but also offer a better understanding of the timing of necessary re-interventions—something that unfortunately occurs throughout life for many of these patients.

Although the numbers of participating patients per center might be rather small, we think it would be a worthwhile and a step towards more personalized medicine for the possibly smallest and weakest patient group.

## 11. Conclusions

Evidence of biomarkers in the CHD population remains heterogeneous, with very limited standardization across studies and small patient cohorts. As a result, while these novel biomarkers are promising, further targeted and large-scale investigations are needed before they can be routinely integrated into clinical practice for CHD management.

## 12. Limitations

This is a non-systematic review with all its limitations, including selection bias, incomplete coverage and author interpretation. Due to the large field and numerous biomarkers tested, we picked a specific group, which our study group will further investigate.

## 13. Further Plans

While cardiac biomarkers hold promise, their role in CHD requires more tailored research to improve diagnostic accuracy and prognostic value. Our research group is exploring the application and prognostic accuracy of emerging biomarkers in pediatric CHD surgery. A study protocol for prospective sampling and clinical testing has been elaborated. Next steps include a systematic review focusing on one of these biomarkers as well as the preliminary results of a pilot study (NCT07029230).

## Data Availability

No new data were created or analyzed in this study. Data sharing is not applicable to this article.
